# Liquid Crystal Lensacons, Logarithmic and Linear Axicons

**DOI:** 10.3390/ma7042593

**Published:** 2014-03-28

**Authors:** José Francisco Algorri, Virginia Urruchi, Braulio García-Cámara, José Manuel Sánchez-Pena

**Affiliations:** Electronic Technology Department, Carlos III University of Madrid, Butarque 15, E28911, Madrid, Spain; E-Mails: vurruchi@ing.uc3m.es (V.U.); brgarcia@ing.uc3m.es (B.G.-C.); jmpena@ing.uc3m.es (J.M.S.-P.)

**Keywords:** axicons, liquid crystal devices, optical trapping

## Abstract

Until now, several attempts have been made to obtain axicons by using liquid crystals. Previous results had always a considerable deviation from the linear response and the resulting aperture is square. In addition, classical fabrications methods are expensive and only produce fixed phase profiles. In this study, a novel structure to obtain tunable axicons with a perfect conical shape and a circular aperture is proposed and theoretically studied. The proposed optical device is based on nematic liquid crystal and phase shifted electrical signals. A simulation program consisted of Finite Elements Method to solve the voltage distribution combined with the Frank-Oseen equation to solve the molecular position of the nematic liquid crystal is employed. This device is totally reconfigurable by using low voltage signals. The focus depth and the position of this one can be controlled electrically.

## 1. Introduction

The term axicon was first proposed by Mcleod in 1954 (from Greek: “axis image”) [1]. In the referred work, a glass cone was first studied to image a point source into a line focus. The main advantage over traditional optical lenses is that axicons produce a field distribution that is proportional to the zero order Bessel function (*J*_0_). In the near field, these beams are characterized by a bright central part surrounded by concentric rings of decreasing intensity. The energy density in the central part of the Bessel beam is high and the focus depth (distance of the preservation) is greater than for Gaussian beams [2]. For this reason, they are also known as non-diffracting Bessel beam (Gaussian beams are broadened and blurred by the diffraction). With the development of optical manufacturing and processing technologies, this type of optical lenses is now commercially available. Despite the usually high fabrication costs, the applications have grown exponentially. First applications were focused on precision alignment for large telescopes [3] and laser machining [4]. After this, negative and positive axicons were used to change the diameter of the Bessel beam ring for ablating corneal material for corneal surgery [5]. In 1992, a scanning optical system based on axicons was proposed [6]; in such a way that now almost every supermarket bar code reader uses axicons. Medical applications have also benefited from the use of axicons, for example: optical coherence tomography [7,8], tissue characterization [9], biophotonics [10,11], among others. Other important applications are optical tweezers [12], atom optics [13], *etc*.

The great number of applications has created an increasing interest in the fabrication of these optical elements. Classical manufacturing methods, using diamond turning from solid materials such as BK7 glass or fused silica, cause them to be expensive. Gradient index (GRIN) axicon has been demonstrated in [14], but the profile has a round axicon tip. Computer-generated holographic optical elements have been also proposed [15] but may require accuracies of nanometers. To overlay these problems, Fresnel axicons were proposed in ref. [16], but they still have the problem of a fixed phase profile.

The parameters defining an axicon are the propagation distance, *z*_max_, the ring spacing, Δρ, and the size of the beam core, *r*_0_. All of these terms proportionally depend on the effective cone angle. This is the main reason of axicons are catalogued by this latter parameter. When the effective cone angle of an axicon can be changed, a total control over the resulting Bessel beam is achieved. However, the variation of the effective cone angle of an axicon is a difficult task. Some systems to make tunable axicons are the fluidic axicon [17] or liquid crystal spatial light modulators (LC-SLM) [18]. Fluidic axicons have a tunable range limited by the refraction index of the fabrication materials. In contrast, the main problems of LC-SLM are the price and the characteristic structure, based on pixels surrounded by opaque areas, causing losses of light efficiency. The pixel resolution causes limitations in the effective cone angle too; the aliasing limits this one to few miliradians [17]. Some works have been researching new ways to create tunable axicons with LC. For example, the generation of axicons with several millimeters of diameter, based on modal control technique, has been proposed in ref. [19]. This technique uses a high resistivity layer to distribute the voltage continuously. In a previous study, we have proposed and demonstrated a structure based on several electrodes and modal control technique to generate an array of four optical elements. This structure can produce four axicons of several millimeters with a high range of tunability [20]. The results obtained have always shown a considerable deviation from the linear response and the resulting aperture is square.

In this study, a structure to obtain tunable axicons with a reconfigurable shape, perfect circular aperture and a high range of tunability, is proposed and theoretically studied. The proposed optical device is based on nematic LC and phase shifted electrical signals. The specific patterned electrode produces a circular aperture. It also reduces the typical phase deviation produced in the edges of the active area when a modal technique is used. A simulation program consisted of Finite Elements Method (FEM) to solve the voltage distribution combined with the Frank-Oseen equation to solve the molecular position of the nematic LC is employed. This device is totally reconfigurable by using low voltage signals. Lensacons, logarithmic axicons and even another type of this kind of optical elements known as fractional axicons, which is a new concept proposed recently in ref. [21], can be obtained. The amplitude of the optical phase shift, as well as the position of this one, can be controlled electrically. This device could contribute to developing new applications and to reduce the fabrication costs of the actual devices.

## 2. Structure and Theoretical Basis

**Figure 1.**
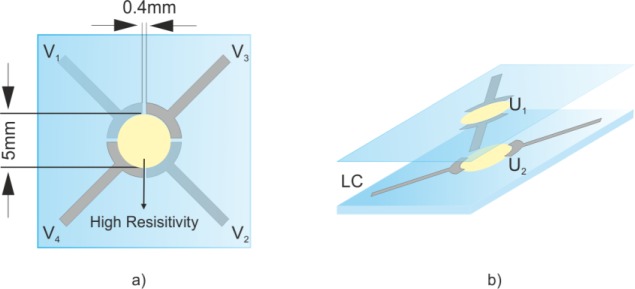
Scheme of the proposed structure. (**a**) Top view and (**b**) 3D diagram of the substrates arrangement. Note drawings are not to scale.

The proposed device comprises two tin-doped indium oxide (ITO) coated glasses separated by spacers of 50 μm. The resulting cavity has to be filled with a MDA 98-1602 nematic LC. The appropriate values of the LC have been obtained from the datasheet issued from the manufacturer (Merck Ltd., Kanagawa, Japan). The design of the electrode pattern at the top and bottom substrate consists of two circular sections of ITO electrodes (Figure 1a). A high resistivity layer covering the ITO electrodes is required. Several materials can be employed, depending on the necessary sheet resistance (*R*_sq_), for instance, Titanium Oxide (TiO_2_), PEDOT, thin films of ITO or Nickel, *etc*. The resistivity of these materials ranges from 0.1 to 10 MΩ/sq. The necessary resistance is inversely proportional to the voltage frequency and directly proportional to the thickness. In this case, a resistivity of 1 MΩ/sq, 1 kHz of voltage frequency and 50 μm of thickness are used in the simulations. A lower *R*_sq_ does not produce any remarkable change, but the power consumption would be higher. Although the thickness is not a critical parameter, the lower the thickness, the lower the optical phase shift, keeping the same phase profile. Substrates are arranged such that their circular areas match with each other. The resulting area is 40 mm^2^. The diameter can be increased if the *R*_sq_ is proportionally decreased. A scheme of the proposed structure, comprising these layers, is shown in Figure 1b. An alignment layer is also deposited over the high resistivity layer (e.g., polyimide). The LC molecules have an elongated shape that causes different molecular polarizabilities between the long and short axis. The effective refractive index depends on the angle between the long axis of the molecules and the linear polarization of the incident light. For this reason, a homogeneous alignment layer, with the same direction as the polarized light, is necessary. After a rubbing process, microgrooves are created in this layer. The main aligning mechanism is based on shear stress induced on the alignment layer [22] and intermolecular interactions between LC and polyimide [23].

The resulting active area is determined by the space between the circular ends of the electrodes. *U*_1_ and *U*_2_ correspond to the voltage distribution in each high-resistivity layer. It is important to mention that the separation distance of 0.4 mm is essential to create perfect axicons and reduce the typical phase deviation produced in the edges of the active area. This specific value is proposed after several optimization tests. If the diameter is increased, this dimension should also be increased in the same proportion. The functionality of this space is established in the next section. The voltage distribution in the active area, which is formed by the two high resistivity layers (*U*_1_ − *U*_2_), results in a second-order two-dimensional coupled partial differential equation (PDE) system.

$$\{\begin{array}{c} \nabla_{s}U_{1}^{2}=R_{\text{sq}1}(G\text{-}j\omega C)\cdot (U_{1}-U_{2})\\ \nabla_{s}U_{2}^{2}=R_{\text{sq}2}(G\text{-}j\omega C)\cdot (U_{2}-U_{1}) \end{array}$$ (1)

where *G* and *C* are distributed conductance and capacitance, respectively, per unit area. *R*_sq1_ and *R*_sq2_ are the sheet resistance of the two high resistivity layers. The solution of this system was experimentally validated in [20]. The solver considers all the possible boundary conditions (Dirichlet and Neumann), mainly determined by the applied voltages. The Frank-Oseen equation in combination with the electrical energy is employed to determine the LC molecular position Equation (2):

$$K_{11}{\left(\nabla \vec{n}\right)}^{2}+K_{22}{\left(\vec{n}\cdot \nabla \times \vec{n}+\frac{2\pi }{\xi }\right)}^{2}+K_{33}{\left|\vec{n}\times \nabla \times \vec{n}\right|}^{2}-\frac{1}{2}\vec{D}\cdot \vec{E}$$ (2)

where $\vec{n}$ (*n_x_*, *n_y_*, *n_z_*) represent the average local orientation of molecules, *K_ii_* are the elastic deformation constants and $\vec{D}$ is the displacement vector and $\vec{E}$ the electric field. The ξ is the pitch of a chiral helicoidal dopant (only for twist purposes). In this case, it is infinite because we use a nematic LC that is just a cholesteric of infinite pitch. As this molecular position also affects the voltage distribution, an iterative process is necessary to determine both the final voltage and molecular position. This molecular distribution generates a gradient in the refraction index which distribution is proportional to the voltage. In our previous work, we demonstrated how the application of complex signals in this type of systems can generate interesting optical modulations. The proposed device in this study goes one step further improving the structure to create axicons with a perfect conical shape, and avoiding the typical defects in the active area edges. Among the structural parameters, the circular patterned and the optimized space between them, are key factors. Moreover, the use of this device to create a new type of optical element, known as fractional axicon, is also theoretically demonstrated.

## 3. Results and Discussion

The simulation program uses the following input parameters about the device structure and the nematic LC features: thickness = 50 μm, *K*_11_ = 13.6 pN, *K*_33_ = 15.7 pN, γ_1_ = 203 mPas, Δ*n* = 0.2666 (*n*_e_ = 1.7779, *n*_o_ = 1.5113), Δ*ε* = 16.2 (ε_e_ = 1.7779, ε_o_ = 1.5113) and *R*_sq1_ = *R*_sq2_ = 1 MΩ/sq, 5° as pretilt angle. The configuration of the device is basically focused on creating three kinds of optical elements: an axicon with a perfect conical shape, which depth of focus and position should be tunable, a lensacon and fractional axicon with the same properties. The driving scheme consists of the application of four electrical signals, with the same frequency (2 kHz) but four different electrical phase shifts (*V*_1_ = 0°, *V*_2_ = 180°, *V*_3_ = 90°, *V*_4_ = 270°) on each electrode. The only configurable parameter is the amplitude of the signals. Additionally, there is necessary an extra signal with different frequency (1 kHz) and variable amplitude (*V*_0_). The electrical phase shift of this signal will be zero and it could be connected to the electrodes by means of an operational amplifier in adder configuration [19]. It creates an offset value in the voltage distribution and eliminate the characteristic zero crossing when phase shifted signals are applied to a modal device.

**Figure 2.**
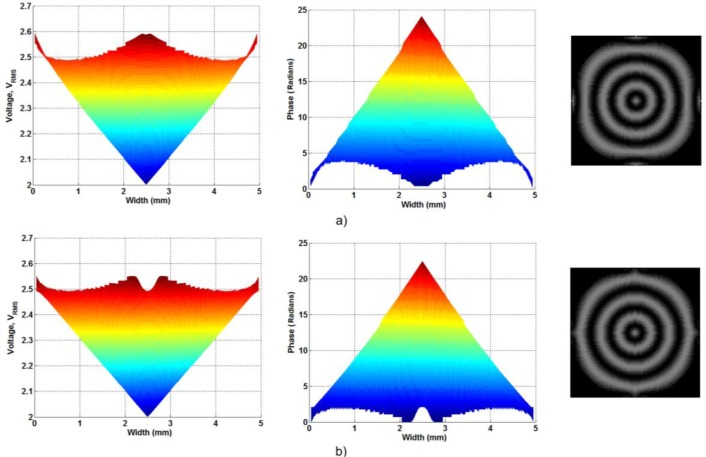
Voltage and optical phase shift distribution in the *XZ* plane, and interference pattern for (**a**) a structure without spaces and (**b**) the proposed structure.

As commented above, the proposed devices based on modal control technique have always square aperture. In this study, we proposed the use of circular electrodes to avoid this problem. Despite this, there is an undesirable effect produced by the modal control configuration; a voltage gradient along the electrodes. It has been observed that the use of little separation distances between electrodes can reduce this effect producing a small gradient voltage at these gaps, but reducing the voltage gradient along the electrodes. The functionality of the proposed spaces is shown in Figure 2.

The use of these spaces solves considerable the problem at the edges of the active area. In the interference pattern, the structure without spaces provoke a semi-square rings in the outer zone of the active area. However, the voltage decreasing produced by the spaces make a considerable lower gradient and the interference pattern reveals a circular ring, even in the outer zone of the active area.

In the following sections three different studies have been taken into account. The first one generates linear axicons and reveals how they are totally reconfigurable in terms of optical phase shift amplitude. This is a clear advantage over commercial axicons that typically produce a fixed phase shift. In Section 3.2 the generation of lenascons and fractional axicons is demonstrated. Finally, the application of this device in optical trapping applications (with a focus movable in *x-y* focal plane) is shown. In combination with the optical phase shift tunability, the position of the optical trap along the z-axis could also be changed.

### 3.1. The Linear Axicon

**Figure 3.**
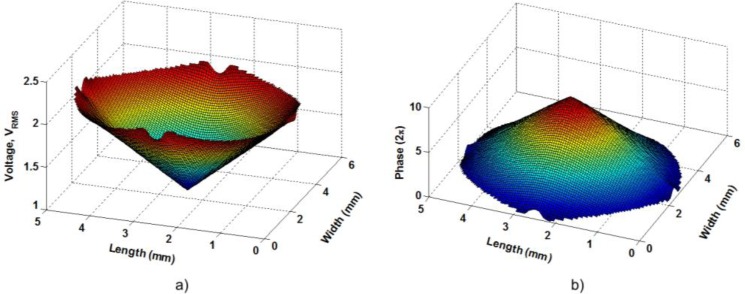
(**a**) Voltage and (**b**) optical phase shift distribution, in three dimensions, of the proposed structure.

A linear axicon is the optical element that produces an optical phase shift with the shape of a perfect cone. Figure 3 shows the voltage distribution and the resulting optical phase shift, of the proposed structure, with the following voltages: *V*_1_ = *V*_2_ = *V*_3_ = *V*_4_ = 1 *V*_rms_ and *V*_0_ = 1.3 *V*_rms_. In this figure, the resulting voltage distribution of Equation (1) has a linear shape.

**Figure 4.**
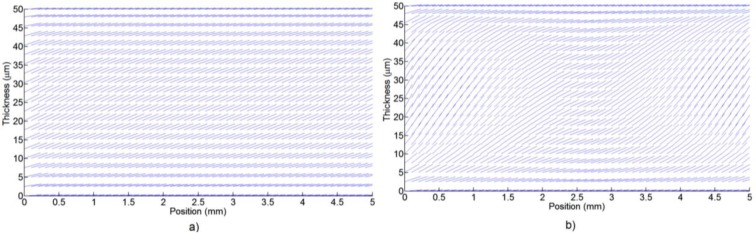
Molecular distribution (*XZ* plane) for (**a**) a low voltage applied to the electrodes (0.1 *V*_rms_); (**b**) and a higher voltage (1.3 *V*_rms_).

However, the LC birefringence, which is determined by the molecular position as a function of voltage, is a non-linear curve. There is a quasi-linear range after the threshold voltage (when the molecules start to move) and before the saturation voltage (when the molecules start to reach the perpendicular position). When a 10% margin from these two limits is used, a linear range can be obtained. Two molecular distributions are shown in Figure 4. Only one plane is selected to clearly see the molecules. Figure 4a uses an offset voltage of 1.3 *V*_rms_ and *V*_1_ = *V*_2_ = *V*_3_ = *V*_4_ = 0.1 *V*_rms_. In contrast, in Figure 4b the molecules are completely oriented and the axicon is formed with the following voltages: *V*_1_ = *V*_2_ = *V*_3_ = *V*_4_ = 1 *V*_rms_ and *V*_0_ = 1.3 *V*_rms_.

It is not straightforward to obtain clear conclusion from these kinds of figures, for the following reason: only one plane is considered and the number of molecules has been reduced. When the light passes through the device the effective refractive index caused by each molecule generates a phase shift between substrates. This is estimated by integration of the molecular effective refractive index over the thickness.

**Figure 5.**
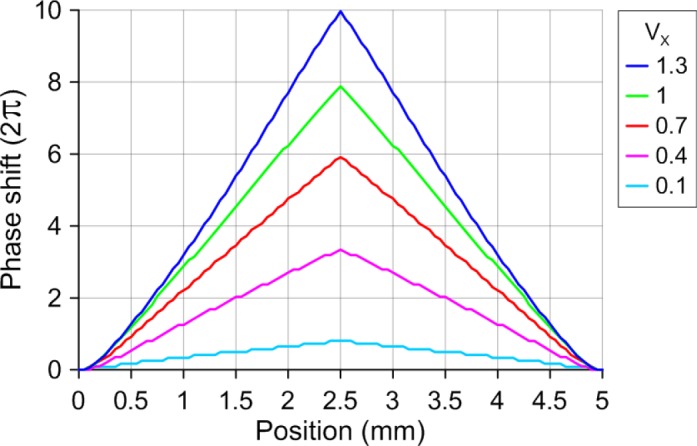
Phase shift profile in the *XZ* plane for several voltages applied to the electrodes.

As can be observed, the voltage distribution produces a linear response in the optical phase shift. For this are necessary small voltage gradients. Despite this, a wide range of tunability can be achieved, as it is shown in Figure 5; only the cone profiles in the *XZ* plane are depicted. The applied voltages are *V*_1_ = *V*_2_ = *V*_3_ = *V*_4_ =*V_x_*·*V*_rms_ and a fixed offset voltage *V*_0_ = 1.3 *V*_rms_.

Due to the high birefringence of MDA-98-1602 the optical phase shift is very high. With a slight variation of 1.2 *V*_rms_, the optical phase shift is tunable from 1.6π radians to 22π radians. In addition, this optical phase shift can be translated to an effective cone angle. Thus, the characteristic parameters could be changed in a broad range.

### 3.2. The Lensacon and Logarithmic Axicon

**Figure 6.**
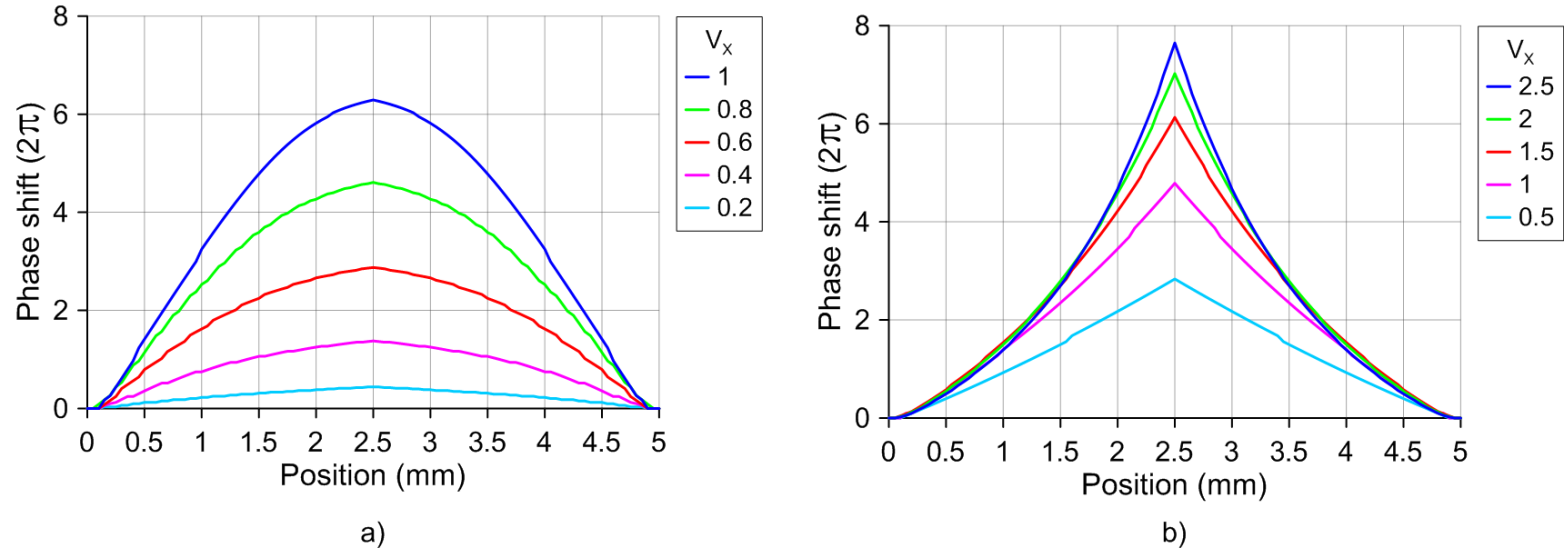
Phase shift profile in the *XZ* plane, for (**a**) lensacons; and (**b**) logarithmic axicons.

**Figure 7.**
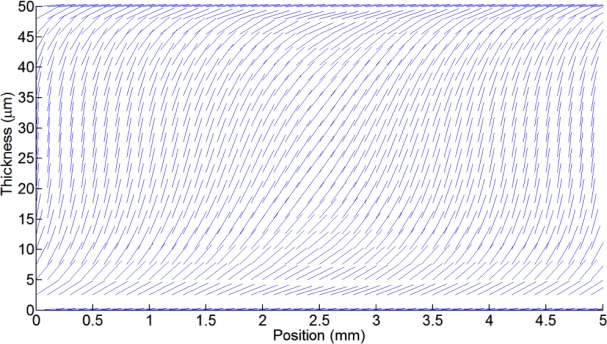
Phase shift profile in the *XZ* plane, for (**a**) lensacons and (**b**) logarithmic axicons.

The non-linear response of the LC birefringence has two characteristic curves. The first one is produced when the electrical energy overcomes the forces that hold the molecules in place, and the molecules start to move. The other one, which has an inverse curvature, is produced when the molecules start to reach the perpendicular position. Taking advantage of these zones, the generation of lensacons [24] and logarithmic axicons [25,26], by using LC is proposed and theoretically demonstrated for the first time. These optical elements are axicons in which their profile phase has a little curvature. In order to create the lensacon, the offset voltage (*V*_0_) has to be smaller than the threshold voltage. In contrast, a voltage smaller to the saturation voltage is required to create logarithmic axicons. Figure 6a shows the phase profile of lensacons, for which the applied voltages are *V*_1_ = *V*_2_ = *V*_3_ = *V*_4_ = *V_x_*·*V*_rms_ and the offset voltage is *V*_0_ = 0.8 *V*_rms_. On the other hand, considering a *V*_0_ = 2.3 *V*_rms_ and *V_x_* = 2.5 *V*_rms_ a logarithmic axicon is observed in Figure 6b. Its molecular distribution is shown in Figure 7.

Changing the voltage of *V*_1_, *V*_2_, *V*_3_ and *V*_4_ (all equal to *V_x_*), these phase profiles could also be tuned. In Figure 6a a tunable lensacon is shown. The optical phase shift can be easily tunable from 0.8π radians to 12π radians, with only a small variation of 0.8 *V*_rms_.

The resulting effect in the case of the logarithmic axicon is a control over the curvature itself. This effect was recently proposed by using diffractive optical elements. This new optical element was called “fractional axicon” (it has a fractional power dependence on the radial coordinate) [21]. In Figure 6b the generation of this optical element simply by low voltage electrical signals is demonstrated.

### 3.3. Moving Focus

The application of optical tweezers based on modal LC devices has considerably reduced functionality over holographic optical traps (HOTs) or the generalized phase contrast (GPC) systems. This is mainly because they can only be used for single or double traps [27]. Despite this, LC devices have a reduced complexity and in consequence they are low-cost and more compact. Other advantages are the analog control (increasing the accuracy) and a larger light efficiency. The proposed device has reduced RMS deviation from the perfect profile. In combination with the experiment proposed in the previous section, the position of the optical trap along the *z*-axis will also be controllable. In order to move the focus in the *x-y* plane, a gradient between electrodes is needed.

**Figure 8.**
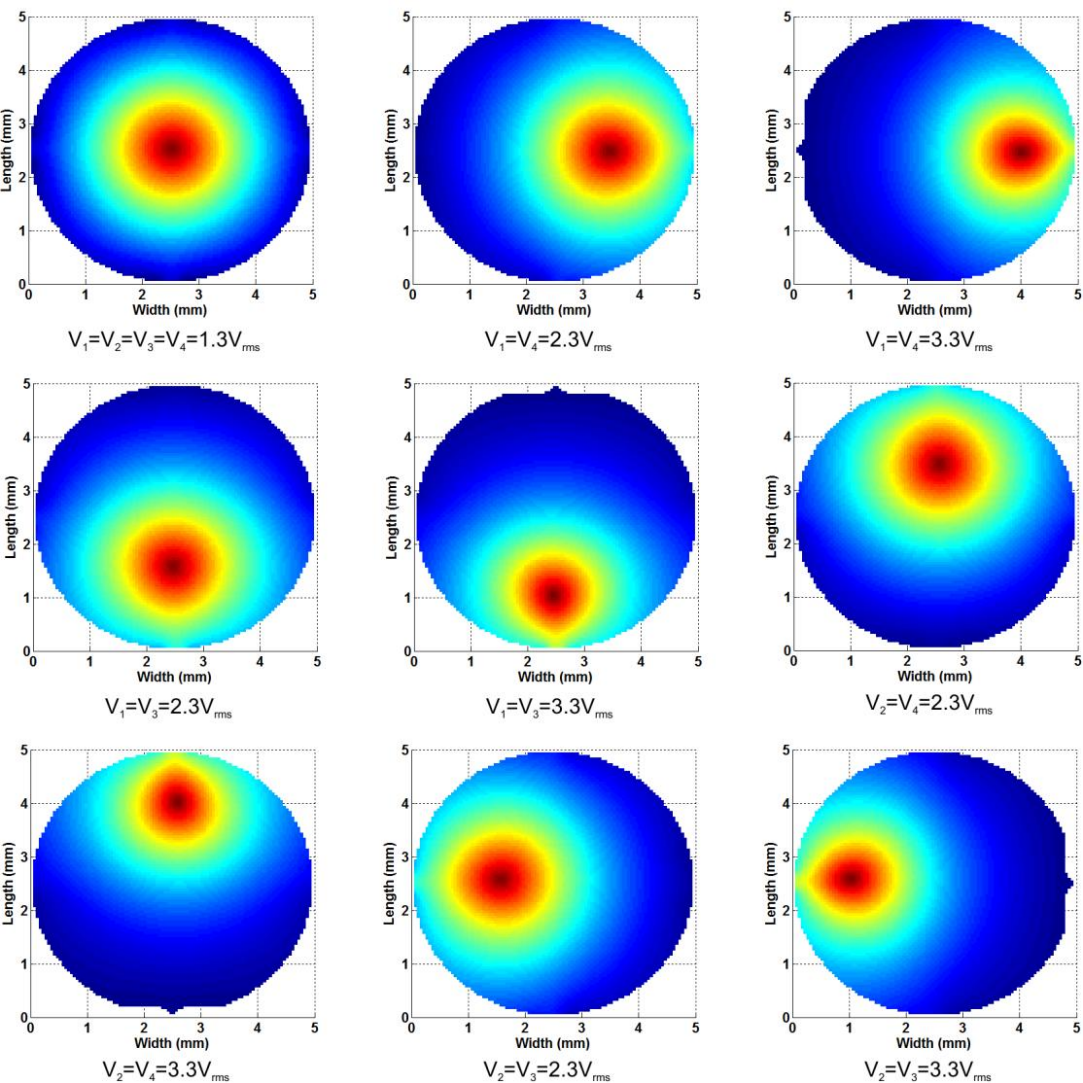
Optical phase shift in the *XY* plane several voltages applied to the electrodes.

The above figures illustrate how the position of the focus can be easily moved in the *x-y* plane. The first image correspond to the same cone as Figure 3 (in *XY* plane) where the applied voltages were *V*_1_ = *V*_2_ = *V*_3_ = *V*_4_ =1.3 *V*_rms_ and *V*_0_ = 1.3 *V*_rms_. Then the voltage is increased only in two electrodes (while the others remains at 1.3 *V*_rms_), as is described in Figure 8. Each pair of voltages move the focus position in one direction, right (*V*_1_, *V*_4_), left (*V*_2_, *V*_3_), up (*V*_2_, *V*_4_), and down (*V*_1_, *V*_3_). In order to change the position of the optical trap along the z-axis, a change in the total voltage (*V_x_*) is required, as was established in the previous section.

The focusing speed of the proposed device is directly related to the rising and decay time of the LC molecules [28],

$$t_{\text{decay}}=\frac{\gamma_{1}}{K_{11}}{\left(\frac{d}{\pi }\right)}^{2}\;t_{\text{rise}}=\frac{\gamma_{1}d^{2}/K_{11}\pi^{2}}{{\left(V/V_{\text{th}}\right)}^{2}-1}$$ (3)

As can be seen these relations are cuadratically proportional to the device thickness. In this case and considering a *V*_th_ = 1.2 *V*_rms_, the decay time will be 3.78 s (switch off the device). If the voltage is increased to 2.3 *V*_rms_, for example, the time necessary for the molecules to reach the new position will be 1.41 s.

## 4. Conclusions

In summary, modal LC axicons offer a simple and low cost solution for the tunable generation of Bessel beams. These ones have several advantages over other reported methods: analog control, high tunability, moving focus in the *x-y* plane and high light efficiency. Unlike other reported LC axicons, the proposed device has a circular aperture that creates perfect cone profiles. The inclusion of some little spaces in the patterned electrode reduces undesirable effects in the edges of the active area (reduced aberrations). For the first time, the generation of perfect axicons, lensacons, logarithm axicons and fractional axicons, by the same structure (totally reconfigurable by low voltages), have been proposed and theoretically demonstrated. We conclude that the simulation tool is essential to design this type of devices because some critical parameters as thickness, resistivity of the deposited layer, frequency and amplitude of the voltage control, can be known before the device fabrication. This device could contribute to developing new applications and to reducing the fabrication costs of the actual devices.
